# A Case–Control Study Investigating Simulated Driving Errors in Ischemic Stroke and Subarachnoid Hemorrhage

**DOI:** 10.3389/fneur.2018.00054

**Published:** 2018-02-13

**Authors:** Megan A. Hird, Kristin A. Vesely, Tahira Tasneem, Gustavo Saposnik, R. Loch Macdonald, Tom A. Schweizer

**Affiliations:** ^1^Neuroscience Research Program, Keenan Research Centre for Biomedical Science, St. Michael’s Hospital, Toronto, ON, Canada; ^2^University of Toronto, Toronto, ON, Canada; ^3^Centre for Hip Health and Mobility, University of British Columbia, Vancouver, BC, Canada; ^4^Stroke Research Unit, Mobility Program, St. Michael’s Hospital, Toronto, ON, Canada; ^5^Medicine, St. Michael’s Hospital, University of Toronto, Toronto, ON, Canada; ^6^Department of Surgery, Neurosurgery Division, University of Toronto, Toronto, ON, Canada; ^7^Institute of Biomaterials and Biomedical Engineering, University of Toronto, Toronto, ON, Canada

**Keywords:** driving performance, simulation, ischemic stroke, subarachnoid hemorrhage, functional outcome

## Abstract

**Background:**

Stroke can affect a variety of cognitive, perceptual, and motor abilities that are important for safe driving. Results of studies assessing post-stroke driving ability are quite variable in the areas and degree of driving impairment among patients. This highlights the need to consider clinical characteristics, including stroke subtype, when assessing driving performance.

**Methods:**

We compared the simulated driving performance of 30 chronic stroke patients (>3 months), including 15 patients with ischemic stroke (IS) and 15 patients with subarachnoid hemorrhage (SAH), and 20 age-matched controls. A preliminary analysis was performed, subdividing IS patients into right (*n* = 8) and left (*n* = 6) hemispheric lesions and SAH patients into middle cerebral artery (MCA, *n* = 5) and anterior communicating artery (*n* = 6) territory. A secondary analysis was conducted to investigate the cognitive correlates of driving.

**Results:**

Nine patients (30%) exhibited impaired simulated driving performance, including four patients with IS (26.7%) and five patients with SAH (33.3%). Both patients with IS (2.3 vs. 0.3, *U* = 76, *p* < 0.05) and SAH (1.5 vs. 0.3, *U* = 45, *p* < 0.001) exhibited difficulty with lane maintenance (% distance out of lane) compared to controls. In addition, patients with IS exhibited difficulty with speed maintenance (% distance over speed limit; 8.9 vs. 4.1, *U* = 81, *p* < 0.05), whereas SAH patients exhibited difficulty with turning performance (total turning errors; 5.4 vs. 1.6, *U* = 39.5, *p* < 0.001). The Trail Making Test (TMT) and Useful Field of View test were significantly associated with lane maintenance among patients with IS (*r*_s_ > 0.6, *p* < 0.05). No cognitive tests showed utility among patients with SAH.

**Conclusion:**

Both IS and SAH exhibited difficulty with lane maintenance. Patients with IS additionally exhibited difficulty with speed maintenance, whereas SAH patients exhibited difficulty with turning performance. Current results support the importance of differentiating between stroke subtypes and considering other important clinical characteristics (e.g., side of lesion, vascular territory) when assessing driving performance and reinforce the importance of physicians discussing driving safety with patients after stroke.

## Introduction

Driving is a highly complex and important daily task. Safe driving requires many abilities, including cognitive, perceptual, and motor function, all of which can be impacted to varying degrees following stroke. Previous research suggests that patients post-stroke are at twice the risk of collision compared to drivers who have not suffered a stroke (1).

Although some patients with stroke do not experience sufficient recovery to drive safely, many regain the ability to drive. Severe visual field deficits, perceptual impairments, and hemiparesis are accepted contraindications to safe driving. However, for patients who experience minor cognitive deficits, including mild attentional and executive dysfunction, that can be compensated for by other cognitive and behavioral functions, determining safe driving ability can be difficult (2). Despite this, there are currently no validated tools or comprehensive guidelines (3) to help physicians assess the driving safety of patients after stroke. Given that driving cessation is associated with numerous negative consequences, it is important to balance patient independence with public and patient safety.

Results of studies assessing post-stroke driving ability (4) are quite variable in the areas and degree of driving impairment among patients (2). One factor contributing to this is the heterogeneity of stroke mechanisms, areas affected, and recovery process of patients. This highlights the need to consider clinical characteristics (5–7) when assessing safe driving ability, including type of stroke.

The aim of our study was to investigate the simulated driving performance of different stroke subtypes—chronic ischemic stroke (IS) and subarachnoid hemorrhage (SAH). Given the different mechanisms of brain injury, as well as cognitive and functional profiles characteristic of IS (8) and SAH (9), it follows that these stroke types might be associated with different driving profiles.

## Materials and Methods

### Participants

Thirty-two patients with stroke, including 15 patients with SAH and 17 patients with IS, and 24 healthy age- and driving experience-matched controls participated in this study. Six (11%) participants experienced simulator sickness, including two patients with IS and four healthy controls, and were unable to complete the current study. This frequency of simulator sickness is consistent with the literature (10–80%) (10). Thus, 30 patients with stroke, including 15 patients with SAH and 15 patients with IS, as well as 20 control participants completed the study.

All patients were recruited from St. Michael’s Hospital, Toronto, ON, Canada. All diagnoses of aneurysmal SAH and IS were confirmed *via* CT or MRI scans. All patients were at least 3 months post-stroke. Any patients with a history of substance abuse, another neurological (e.g., Alzheimer’s disease, and so on) or psychiatric condition (e.g., bipolar disorder, and so on), motor deficits that would prohibit the manipulation of the equipment, or visual deficits that failed to meet the Ontario Ministry of Transportation standards for driving were excluded. All patients currently held or previously held a valid driver’s license immediately preceding the stroke.

Age- and driving experience-matched healthy control participants were recruited through the community and held a valid driver’s license. Control participants had no history of neurological or psychiatric condition, no history of substance use, and no significant visual or motor abnormalities. Any control participant who scored <26 on the Montreal Cognitive Assessment (MoCA) (11) was excluded from the analyses.

This study was carried out in accordance with the recommendations of the Research Ethics Board at St. Michael’s Hospital with written informed consent from all participants. All participants gave written informed consent in accordance with the Declaration of Helsinki and the protocol was approved by the Research Ethics Board at St. Michael’s Hospital.

### Driving Simulation

A portable driving simulator (Logitech G25 model, STISIM Drive^®^) was used to assess driving performance. The set-up included a steering wheel, accelerator pedal, brake pedal, and signaling system. During the experimental session, participants drove through a city driving scenario with various conditions—straight driving, right and left turns, and left turns with oncoming traffic. At intersections with oncoming traffic, participants need to judge when it is safe to turn in order to avoid other vehicles and pedestrians, which requires greater cognitive effort (12). Variables of interest included the following: collisions, centerline crossings, road edge excursions, stop signs missed, speed exceedances, total driving errors (i.e., the sum of all individual errors), percentage distance out of the legal driving lane and over the posted speed limit, speed variability (SD in speed), and lane variability [standard deviation in lane position (SDLP)]. Patients were classified as “impaired” if the number of total driving errors committed was three SDs above the mean number of errors committed by healthy control drivers.

### Cognitive Tests

Cognitive tests that were administered to participants included the following: the MoCA (11), the Trail Making Test Part A (TMT-A) and Part B (TMT-B) (13), and the Useful Field of View test (UFOV) (14). The MoCA is a quick screening tool for cognitive dysfunction and was used to screen healthy control participants for any underlying cognitive impairment. The TMT is a measure of attention, speed, and mental flexibility. The UFOV measures vision and visual attention. Both the TMT and UFOV have been widely explored in the stroke and driving literature (15).

### Statistical Analyses

Data collected from patients with stroke and healthy controls were compared using the non-parametric Mann–Whitney *U* test and Kruskal–Wallis *H* test. *Post hoc* testing was conducted with Bonferroni corrections for two comparisons—IS stroke vs. healthy controls and SAH vs. healthy controls.

A preliminary analysis was performed to investigate the effect of clinical characteristics on driving performance with the IS and SAH groups. Kruskal–Wallis *H* test was used to compare the performance of (1) right and left hemispheric lesions among the IS group and (2) anterior communicating artery and middle cerebral artery (MCA) aneurysms for the SAH group with the performance of healthy control drivers across select variables of interest. A secondary analysis investigating the association between cognitive scores (MoCA, TMT time, UFOV subscores) as well as duration since stroke onset and driving performance (total errors, turning errors, and percentage distance out of the lane and over the speed limit) was conducted using the Spearman’s rank correlation with bootstrapping.

## Results

There were no significant differences between patients with stroke and healthy controls on most demographic variables, including age, sex, driving experience (years and hours of driving per week), and self-reported accidents (*p* > 0.05); however, patients with SAH had a significantly lower education compared to healthy controls and patients with IS (*p* < 0.001; see Table 1 for Demographic and Cognitive Scores). Patients with IS and SAH scored significantly worse on the MoCA and TMT-A compared to healthy controls (*p* < 0.01). Patients with SAH additionally scored significantly worse on TMT-B compared to healthy controls (*p* < 0.05).

**Table 1:** Demographic and cognitive scores all stroke patients, including ischemic stroke (IS) patients and SAH patients, and healthy age-matched controls.

	Healthy controls (*n* = 20)	All stroke patients (*n* = 30)	IS patients (*n* = 15)	SAH patients (*n* = 15)	*p*-Value
Age	62 (10.6)	59.2 (11.5)	60.3 (12.3)	58.1 (10.8)	0.464
Gender, male *n* (%)	15 (75%)	18 (60%)	12 (80%)	6 (40%)	0.068
Education	17.0 (2.3)^a^	15.0 (2.2)	16.1 (1.9)^a^	14.0 (2.0)^b^	**0.001**
Driving experience, years	41.9 (12.8)	35.3 (17.6)	39.2 (17.7)	31.7 (17.2)	0.170
Driving experience, h/week	7.1 (6.6)	12.7 (14.4)	9.2 (11.0)	16.5 (16.9)	0.075
Self-reported accidents	1.9 (2.4)	0.9 (1.0)	0.9 (0.8)	0.9 (1.3)	0.226
MoCA, total	27.9 (1.2)^a^	25.3 (3.5)	26.2 (2.5)^b^	24.4 (4.2)^b^	**0.007**
TMT-A time	22.2 (4.8)^a^	37.1 (22.3)	39.8 (29.1)^b^	34.3 (13.0)^b^	**0.006**
TMT-B time	50.0 (22.2)^a^	90.5 (64.6)	93.1 (75.8)^a,b^	87.7 (52.7)^b^	**0.017**
UFOV processing speed	23.9 (16.3)	28.5 (20.5)	25.6 (14.7)	31.8 (26.1)	0.465
UFOV divided attention	39.0 (32.5)	149.3 (152.2)	170.0 (174.1)	122.4 (121.5)	0.052
UFOV selective attention	142.7 (71.2)	207.7 (149.4)	206.1 (152.3)	210.7 (155.9)	0.235

*Values are reported in mean ± SD format*.

*bolded values indicate analyses that are statistically significant*.

*n, number of participants; SAH, subarachnoid hemorrhage; TMT, Trail Making Test; UFOV, Useful Field of View; MoCA, Montreal Cognitive Assessment*.

*p-values reported for one-way ANOVA (when statistical assumptions were met) and Kruskal–Wallis test (when statistical assumptions were violated) analyses (i.e., healthy controls vs. IS vs. SAH)*.

*^a,b^Superscripts denote whether a significant difference emerged between groups (i.e., shared superscripts indicated no significant difference at p = 0.05)*.

The average duration between stroke onset and time of testing was 34 months for IS patients and 22.5 months for SAH patients (Table 2). All patients with IS scored in the mild to moderate range (range = 0–10) of the National Institute of Health Stroke Scale (NIHSS) (16) at admission and in the mild range (range = 0–5) at hospital discharge. All patients with SAH were considered to have “good grade” SAH (17) and scored between 1 and 3 on the World Federation of Neurological Societies SAH (WFNS) classification.

**Table 2:** Clinical characteristics of ischemic stroke (IS) patients and SAH patients.

	Frequency or mean ± SD
IS patients (*n* = 15)	
Time since onset in months, mean ± SD (range)	34.0 ± 23.9 (3.2–90.1)
Side of lesion	
Left	6
Right	8
Bilateral	1
Vascular territory, frequency	
MCA	6
PCA	3
AChA	1
Cerebellar	1
Unknown	4
Lesion location, frequency	
Frontal	1
Temporal/parietal	1
Parietal/corona radiata	1
Occipital	1
Basal ganglia	3
Hippocampus	1
Insula	1
Thalamus	2
Corpus collasum/anterior cinglulate	1
Cerebellum	1
Internal capsule	1
Frontal/temporal/parietal/insula	1
NIHSS Score at admission, mean ± SD (range)	3.1 ± 3.8 (0–10)
NIHSS Score at discharge, mean ± SD (range)	0.9 ± 1.6 (0–5)
NIHSS Score at admission, frequency	
0	5
1	1
2	1
3	2
4	1
5–10^a^	3
Unknown	2
SAH patients (*n* = 15)	
Time since onset, mean ± SD (range)	22.5 ± 13.7 (5.8–60.1)
Aneurysm location, frequency	
Acomm	6
Pcomm	2
MCA	5
ICA	1
Basilar	1
WFNS classification, mean ± SD (range)	1.6 ± 0.8 (1–3)
WFNS classification, frequency	
1	9
2	3
3	3

*n, number of participants; NIHSS, National Institute of Health Stroke Scale; SAH, subarachnoid hemorrhage; WFNS, World Federation of Neurological Societies SAH*.

*^a^Of the patients who scored in the 5–10 range on the NIHSS, one patient scored an “8” and two patients scored a “10” on admission*.

### Driving Performance of Individuals after Stroke

Nine patients (30%) exhibited driving impairment, committing more than three SDs above the mean number of errors committed by healthy control drivers (>28 total driving errors). No control drivers were classified as impaired. Patients with stroke (IS + SAH) committed significantly more total driving errors (21.2 vs. 10.9, *U* = 137.5, *p* = 0.001), centerline crossings (5.7 vs. 1.6, *U* = 142.5, *p* < 0.005), road edge excursions (1.9 vs. 0.4, *U* = 207.5, *p* < 0.05), and speed exceedances (10.8 vs. 6.8, *U* = 197, *p* < 0.05) compared to healthy controls (Table 3). Stroke patients spent a significantly greater distance of the run out of the legal driving lane (1.9 vs. 0.3, *U* = 121, *p* < 0.001) and over the posted speed limit (9.0 vs. 4.1, *U* = 183.5, *p* < 0.05). Patients with stroke committed significantly more turning errors (i.e., sum of collisions and lane deviations across right turns, left turns, and left turns with traffic; 4.6 vs. 1.6, *U* = 127.5, *p* = 0.001) as well as right turns (2.3 vs. 0.8, *U* = 183.5, *p* < 0.05) and left turns with oncoming traffic (1.6 vs. 0.65, *U* = 178, *p* < 0.05) compared to healthy controls.

**Table 3:** Simulated driving results of all stroke patients, including ischemic stroke (IS) patients and SAH patients, and healthy age-matched controls.

	Healthy controls (*n* = 20)	All stroke patients (*n* = 30)	IS patients (*n* = 15)	SAH patients (*n* = 15)	*p*-Value
Collisions	0.2 ± 0.4	1.1 ± 2.4	0.8 ± 1.6	1.5 ± 3.0	0.074^a^
					0.147^b^

Speed exceedances	6.8 ± 5.8	10.8 ± 6.7	10.9 ± 6.0	10.7 ± 7.7	**0.041^a^**
					0.115^b^

Centerline crossings	1.6 ± 1.7	5.7 ± 6.8	6.1 ± 8.5	5.3 ± 4.8	**0.002^a^**
					**0.004^b^**

Road edge excursions	0.4 ± 0.8	1.9 ± 2.7	1.4 ± 2.7	2.5 ± 2.7	**0.043^a^**
					**0.014^b^**

Stop signs missed	1.7 ± 2.1	1.7 ± 2.1	2.3 ± 2.5	1.1 ± 1.5	0.812^a^
					0.323^b^

Total errors^c^	10.9 ± 5.7	21.2 ± 13.9	21.5 ± 13.8	21.0 ± 14.5	**0.001^a^**
					**0.005^b^**

% Distance out of legal driving lane	0.3 ± 0.3	1.9 ± 2.8	2.3 ± 3.7	1.5 ± 1.4	**<0.001^a^**
					**0.001^b^**

% Distance over speed limit	4.1 ± 4.3	9.0 ± 9.5	8.9 ± 9.6	9.1 ± 9.7	**0.021^a^**
					0.060^b^

SDLP	4.3 ± 0.2	4.2 ± 0.3	4.3 ± 0.3	4.2 ± 0.3	0.430^a^
					0.316^b^

SD in speed	19.9 ± 2.2	20.9 ± 2.5	20.6 ± 2.0	21.2 ± 3.0	0.129^a^
					0.257^b^

Right turn errors^d^	0.8 ± 1.1	2.3 ± 2.3	2.1 ± 2.1	2.5 ± 2.6	**0.016^a^**
					0.054^b^

Left turn errors^d^^,^^e^	0.1 ± 0.4	0.6 ± 1.0	0.2 ± 0.6	0.9 ± 1.2	0.153^a^
					0.027^b^

Left turn + traffic errors^d^	0.6 ± 0.7	1.6 ± 1.7	1.5 ± 2.0	1.7 ± 1.4	**0.011^a^**
					**0.023^b^**
Total turning errors^d,f^	1.6 ± 1.4	4.6 ± 3.6	3.9 ± 3.6	5.4 ± 3.6	**0.001^a^**
					**0.001^b^**

*Values are reported in mean ± SD format*.

*bolded values indicate analyses that are statistically significant*.

*n, number of participants; SDLP, standard deviation in lane position*.

*^a^p-values reported for all stroke patients vs. healthy control comparison (Mann–Whitney U test)*.

*^b^p-values reported from Kruskal–Wallis test analyses (IS stroke vs. SAH vs. healthy control comparison)*.

*^c^Total errors include: sum of collisions, speed exceedances, centerline crossings, road edge excursions, stop signs missed*.

*^d^Turning errors include collisions, centerline crossings, and road edge excursions*.

*^e^Kruskal–Wallis test was significant for left turns across stroke subtypes and controls, but results were no longer significant after the Bonferroni correction. The number of right turns is greater than the number of left turns and left turns with traffic, which is why the number of errors committed is greatest during this condition*.

*^f^Total turning errors: sum of errors committed during right turns, left turns, and left turns with traffic*.

### Driving Performance of Individuals after SAH and IS

Of the nine patients who were classified as “impaired,” four (26.7%) were diagnosed with IS and five (33.3%) were diagnosed with SAH. The clinical characteristics of the impaired SAH and IS patients are reported in Tables 4 and 5, respectively. The nine patients who exhibited impaired driving performance in the current were quite variable in presentation, and no strong clinical patterns emerged.

**Table 4:** Clinical characteristics of patients with SAH who were impaired on the driving simulation assessment.

	Age (years)	Sex (M/F)	Time since onset (months)	Location	WFNS	MoCA Score
SAH-1	76	F	18.2	R PComm	2	17
SAH-2	41	F	27.2	L ICA	1	28
SAH-3	41	F	22.3	R MCA	1	17
SAH-4	44	F	10.9	L MCA	1	28
SAH-5	50	M	29.2	AComm	3	29

*F, female; ICA, internal carotid artery; M, male; MCA, middle cerebral artery; MoCA, Montreal Cognitive Assessment; SAH, subarachnoid hemorrhage; WFNS, World Federation of Neurological Societies SAH*.

**Table 5:** Clinical characteristics of patients with ischemic stroke (IS) who were impaired on the driving simulation assessment.

	Age (years)	Sex (M/F)	Time since onset (months)	Side of infarct	Vascular territory	NIHSS at admission	NIHSS at discharge	MoCA score
IS-1	68	F	51.4	L	MCA	0	0	26
IS-2	71	M	24.8	R	PCA	10	0	26
IS-3	63	M	51.4	R	MCA	10	5	27
IS-4	67	F	9.9	R	Internal capsule	0	0	27

*F, female; M, male; MCA, middle cerebral artery; MoCA, Montreal Cognitive Assessment; NIHSS, National Institute of Health Stroke Scale; PCA, posterior cerebral artery*.

Subarachnoid hemorrhage patients committed significantly more total driving errors (21.0 vs. 10.9, *U* = 78, *p* < 0.05), centerline crossings (5.3 vs. 1.6, *U* = 48.5, *p* < 0.001) as well as road edge excursions (2.5 vs. 0.4, *U* = 68.5, *p* = 0.01) and spent a significantly greater percentage distance out of the legal driving lane (1.5 vs. 0.3, *U* = 45, *p* < 0.001) compared to controls. IS patients committed more total driving errors (21.5 vs. 10.9, *U* = 59.5, *p* < 0.05) and spent a significantly greater percentage distance out of the legal driving lane (2.3 vs. 0.3, *U* = 76, *p* < 0.05) and over the speed limit (8.9 vs. 4.1, *U* = 81, *p* < 0.05) compared to healthy controls. SAH patients committed significantly more overall turning errors (5.4 vs. 1.6, *U* = 39.5, *p* < 0.001) as well as left turns with traffic (1.7 vs. 0.65, *U* = 76, *p* < 0.05) compared to controls. Although SAH patients committed more left turn errors compared to controls, results did not maintain significance after correcting for multiple comparisons.

Given the heterogeneous distribution of sex among patients with SAH and IS, we compared the driving performance of males and females among all stroke patients, patients with IS, and patients with SAH. No significant differences emerged for sex across these groups, including lane deviations, total driving errors, left turns with traffic, total turning errors, percentage distance out of the legal driving lane, and percentage distance over the speed limit (*p* > 0.05).

#### Side of Lesion and Driving Performance in IS Patients

A preliminary analysis was performed to investigate differences in driving performance among IS patients with right (*n* = 8) and left (*n* = 6) sided lesions across select variables of interest. No significant differences emerged between controls and IS patients with left-sided lesions; however, patients with right-sided lesions committed significantly more total driving errors (24.6 vs. 10.9, *U* = 27.5, *p* < 0.05) and spent a significantly greater distance out of the legal driving lane (3.3 vs. 0.3, *U* = 26.0, *p* = 0.01) compared to healthy control drivers. No cognitive differences emerged for left-sided lesions and control participants, whereas patients with right-sided lesions performed significantly worse on the TMT-A, TMT-B, MoCA, and UFOV divided attention (Table 6).

**Table 6:** Simulated driving and cognitive results for IS with right and left-sided lesions and healthy age-matched controls.

	Healthy controls (*n* = 20)	Left-sided lesion (*n* = 6)	Right-sided lesion (*n* = 8)	*p*-Value
Total errors^a^	10.9 ± 5.7	17.3 ± 7.9	24.6 ± 17.7	**0.016**
Centerline crossings	1.6 ± 1.7	3.0 ± 2.2	8.6 ± 11.1	0.119
% Distance out of legal driving lane	0.3 ± 0.3	0.3 ± 0.2	3.3 ± 4.6	**0.018**
% Distance over speed limit	4.1 ± 4.3	6.8 ± 5.9	11.1 ± 12.1	0.088
LT+T errors	0.6 ± 0.7	1.3 ± 0.8	1.6 ± 2.7	0.216
Total turning errors^b^	1.6 ± 1.4	3.2 ± 2.5	4.2 ± 4.5	0.065
MoCA	27.9 (1.2)	27.7 ± 1.5	25.7 ± 2.2	**0.028**
TMT-A time	22.2 (4.8)	25.1 ± 7.3	42.1 ± 27.9	**0.033**
TMT-B time	50.0 (22.2)	47.3 ± 26.0	99.1 ± 42.7	**0.018**
UFOV processing speed	23.9 (16.3)	17.6 ± 1.3	29.3 ± 18.2	0.066
UFOV divided attention	39.0 (32.5)	80.2 ± 132.6	187.0 ± 153.9	**0.003**
UFOV selective attention	142.7 (71.2)	131.6 ± 118.9	229.3 ± 155.9	0.238

*Values are reported in mean ± SD format*.

*bolded values indicate analyses that are statistically significant*.

*n, number of participants; TMT, Trail Making Test; UFOV, Useful Field of View*.

*p-values reported for Kruskal–Wallis test analyses (i.e., healthy controls vs. left-sided lesion vs. right-sided lesion). *Post hoc* analyses results revealed significant differences between controls and patients with right-sided lesions only*.

*^a^Total errors include: sum of collisions, speed exceedances, centerline crossings, road edge excursions, stop signs missed*.

*^b^Total turning errors: sum of errors committed during right turns, left turns, and left turns with traffic*.

#### Anterior Communicating Artery and MCA Aneurysms and Driving Performance

A second preliminary analysis was performed to investigate differences in driving performance among SAH patients with anterior communicating artery (*n* = 6) and MCA (*n* = 5) aneurysms across the same select variables of interest. No significant differences emerged between controls and patients with anterior communicating artery aneurysms. Patients with MCA aneurysms spent a significantly greater percentage out of the legal driving lane compared to controls (1.0 vs. 0.3, *U* = 26.0, *p* = 0.01). Furthermore, patients with MCA aneurysms performed significantly worse on the MoCA and on the TMT-A (Table 7).

**Table 7:** Simulated driving and cognitive results for SAH patients with anterior communicating artery aneurysms and MCA aneurysms and healthy age-matched controls.

	Healthy controls (*n* = 20)	MCA (*n* = 5)	AComm (*n* = 6)	*p*-Value
Total errors^a^	10.9 ± 5.7	17.0 ± 12.1	15.8 ± 7.8	0.275
Centerline crossings	1.6 ± 1.7	3.2 ± 1.3	3.0 ± 1.7	0.030^c^
% Distance out of legal driving lane	0.3 ± 0.3	1.0 ± 1.0	0.9 ± 0.7	**0.024**
% Distance over speed limit	4.1 ± 4.3	6.1 ± 7.7	5.9 ± 5.5	0.630
LT + T errors	0.6 ± 0.7	1.8 ± 1.3	1.3 ± 1.2	0.125
Total turning errors^b^	1.6 ± 1.4	5.0 ± 3.7	3.0 ± 0	0.011
MoCA	27.9 ± 1.2	23.6 ± 4.6	25.7 ± 4.0	**0.045**
TMT-A time	22.2 ± 4.8	43.6 ± 8.7	23.6 ± 2.2	**0.002**
TMT-B time	50.0 ± 22.2	95.8 ± 60.5	61.0 ± 22.6	0.064
UFOV processing speed	23.9 ± 16.3	45.0 ± 39.6	20.8 ± 6.9	0.705
UFOV divided attention	39.0 ± 32.5	228.0 ± 35.3	64.2 ± 49.3	0.055
UFOV selective attention	142.7 ± 71.2	420.5 ± 23.3	116.0 ± 73.2	0.056

*Values are reported in mean ± SD format*.

*bolded values indicate analyses that are statistically significant*.

*Acomm, anterior communicating artery; MCA, middle cerebral artery; n, number of participants; SAH, subarachnoid hemorrhage; TMT, Trail Making Test; UFOV, Useful Field of View*.

*p Values reported for Kruskal–Wallis test analyses (i.e., healthy controls vs. MCA vs. anterior communicating artery). Post hoc analyses results revealed significant differences between controls and patients with MCA aneurysms only*.

*^a^Total errors include sum of collisions, speed exceedances, centerline crossings, road edge excursions, and stop signs missed*.

*^b^Total turning errors: sum of errors committed during right turns, left turns, and left turns with traffic*.

*^c^Not significant after post hoc testing with Bonferroni correction*.

### Correlations between Driving Errors and Cognitive Test Scores

No significant correlations emerged between any of the driving variables of interest and cognitive test scores for all patients with stroke (SAH + IS). For patients with IS, TMT-A time [*r*_s_ = 0.680, 95% CI = (0.172, 0.947), *p* < 0.05], TMT-B time [*r*_s_ = 0.608, 95% CI = (0.042, 0.966), *p* < 0.05], UFOV divided attention [*r*_s_ = 0.729, 95% CI = (0.278, 0.942), *p* < 0.01], and UFOV selective attention [*r*_s_ = 0.712, 95% CI = (0.280, 0.896), *p* < 0.01] were significantly correlated with percentage distance out of the driving lane. Duration since stroke onset (months) was not correlated with any driving variable of interest for all patients with stroke, SAH only, or IS only. Cognitive correlation results are reported in Table 8.

**Table 8:** Correlations between cognitive scores and driving errors in all stroke patients, including ischemic stroke (IS) patients and SAH patients, and healthy age-matched controls.

	All stroke patients (*n* = 30)	IS patients (*n* = 15)	SAH patients (*n* = 15)
**Total errors**			
Stroke duration (months)	−0.13 (−0.341, 0.315)	−0.55 (−0.652, 0.444)	0.088 (−0.322, 0.515)
MoCA (total)	0.176 (−0.224, 0.570)	0.87 (−0.458, 0.691)	0.144 (−0.750, 0.830)
TMT-A time	−0.072 (−0.573, 0.479)	0.354 (−0.266, 0.766)	−0.607 (−1.00, 0.400)
TMT-B time	0.027 (−0.393, 0.425)	0.130 (−0.407, 0.617)	−0.536 (−0.923, 0.556)
UFOV processing speed	−0.015 (−0.453, 0.476)	0.253 (−0.465, 0.761)	−0.236 (−0.917, 0.704)
UFOV divided attention	−0.066 (−0.527, 0.483)	0.415 (−0.159, 0.788)	−0.883 (−1.00, −0.412)^†^
UFOV selective attention	−0.053 (−0.501, 0.472)	0.477 (−0.203, 0.825)	−0.714 (−1.00, −0.059)

**Turning errors**			
Stroke duration (months)	−0.149 (−0.512, 0.203)	−0.144 (−0.683, 0.449)	0.070 (−0.366, 0.491)
MoCA (total)	−0.079 (−0.540, 0.459)	−0.019 (−0.664, 0.727)	−0.60 (−0.837, 0.882)
TMT-A time	0.175 (−0.408, 0.695)	0.392 (−0.248, 0.852)	−0.217 (−0.970, 0.832)
TMT-B time	0.188 (−0.287, 0.628)	0.229 (−0.422, 0.841)	0.296 (−0.508, 0.882)
UFOV processing speed	0.182 (−0.273, 0.585)	0.344 (−0.258, 0.764)	−0.337 (−0.996, 0.928)
UFOV divided attention	0.175 (−0.331, 0.668)	0.461 (−0.229, 0.955)	−0.646 (−1.00, −0.029)
UFOV selective attention	0.177 (−0.277, 0.591)	0.373 (−0.309, 0.909)	−0.433 (−1.00, 0.454)

**% Distance out of lane**			
Stroke duration (months)	−0.300 (−0.597, 0.045)	−0.334 (−0.750, 0.245)	−0.246 (−0.669, 0.275)
MoCA (total)	−0.250 (−0.689, 0.296)	−0.424 (−0.890, 0.333)	−0.018 (−0.837, 0.765)
TMT-A time	0.256 (−0.287, 0.724)	**0.680** (**0.172, 0.947**)*	−0.429 (−1.00, 0.647)
TMT-B time	0.326 (−0.135, 0.715)	**0.608** (**0.042, 0.966**)*	−0.393 (−0.882, 0.686)
UFOV processing speed	0.297 (−0.256, 0.698)	0.565 (−0.254, 0.942)	−0.099 (−0.828, 0.817)
UFOV divided attention	0.251 (−0.251, 0.685)	**0.729** (**0.278, 0.942**)^†^	−0.829 (−1.00, −0.245)*
UFOV selective attention	0.242 (−0.209, 0.625)	**0.712** (**0.290, 0.896**)^†^	−0.607 (−1.00, 0.59)

**% Distance over speed**			
Stroke duration (months)	0.218 (−0.130, 0.521)	0.178 (−0.445, 0.667)	0.200 (−0.253, 0.607)
MoCA (total)	0.092 (−0.303, 0.497)	−0.84 (−0.559, 0.482)	0.054 (−0.959, 0.882)
TMT-A time	−0.281 (−0.679, 0.169)	0.038 (−0.583, 0.528)	−0.750 (−1.00, 0.176)
TMT-B time	−0.080 (−0.331, 0.450)	−0.143 (−0.689, 0.464)	−0.750 (−1.00, 0.125)
UFOV processing speed	−0.194 (−0.618, 0.255)	−0.048 (−0.642, 0.565)	−0.433 (−0.899, 0.449)
UFOV divided attention	−0.138 (−0.601, 0.361)	0.102 (−0.525, 0.643)	−0.595 (−1.00, 0.169)
UFOV selective attention	−0.217 (−0.589, 0.235)	0.074 (−0.536, 0.618)	−0.643 (−1.00, 0.176)

*Values are reported in *r*_s_ (95% confidence interval) format*.

*MoCA, Montreal Cognitive Assessment; SAH, subarachnoid hemorrhage; TMT, Trail Making Test; UFOV, Useful Field of View*.

*p<0.05.

*^†^p<0.01*.

## Discussion

Despite the high prevalence of cognitive, perceptual, and motor deficits after stroke, patients are not consistently provided with driving advice from physicians or any form of assessment to evaluate their ability to drive safely (18). To our knowledge, this study represents the first to evaluate the driving performance of two different stroke types with different mechanisms of brain injury—IS and SAH. In the present study, 30% of patients, including 26.7% of IS and 33.3% of SAH patients exhibited impairment in overall simulated driving performance. Both patients with IS and SAH exhibited difficulty with lane maintenance. Patients with IS additionally exhibited difficulty with speed maintenance, whereas patients with SAH exhibited difficulty with turning performance.

On a group level, stroke patients committed approximately twice as many total driving errors and over three times as many centerline crossings compared to healthy controls. Previous studies assessing driving ability after stroke reported variable results, with some on-road and simulated driving studies reporting that stroke patients are at an increased risk of impairment, including lane positioning and overall performance, whereas others report little to no impairment in these areas (2). One factor contributing to these inconsistencies is the variable methodology employed across studies, including the heterogeneity of stroke (e.g., the underlying stroke mechanism, affected brain regions, and pathways), study design, and driving performance protocols (2, 5, 6).

Both IS and SAH patients similarly exhibited difficulty with lane maintenance (IS: percentage distance out of driving lane; SAH: percentage distance out of driving lane, number of lane deviations). This suggests that, regardless of subtype, patients after stroke may be at risk for impairment in lane control. Furthermore, differences in driving profiles emerged for the two subtypes, highlighting the importance of evaluating the driving ability of various stroke subtypes separately. Specifically, IS patients additionally demonstrated difficulty with speed control (percentage distance over the speed limit), whereas SAH patients exhibited difficulty with turning behavior, particularly during more cognitively demanding left turns with oncoming traffic.

A previous study conducted by Devos and colleagues (5) supported the importance of accounting for clinical characteristics as well as assessing lane positioning and performance during cognitively demanding aspects of driving after stroke. Specifically, lateral lane position change and tasks requiring higher order cognitive processing (e.g., understanding, insight, and quality of traffic participation) emerged as the best predictors of on-road test decision (5). Furthermore, different items emerged as the best predictors of performance for right and left hemisphere lesions. These results combined with current results reinforce the importance of (1) evaluating lane maintenance and cognitively demanding aspects of driving, including turning and (2) accounting for various stroke characteristics (e.g., lateralization, stroke type, etc.) when assessing the driving performance of patients after stroke.

A preliminary analysis was performed to investigate the utility of select clinical variables in characterizing the driving performance of IS and SAH. Specifically, we compared the driving performance of healthy controls and stroke patients by stratifying by side of lesion for IS patients and aneurysm territories for SAH patients (anterior communicating artery and MCA). Given the low sample size of the current study, this preliminary analysis was limited to these variables. IS with right-sided lesions (*n* = 8) committed significantly more total driving errors and demonstrated difficulty with lane maintenance (percentage distance out of the lane) compared to healthy control drivers. Furthermore, IS patients with right-sided lesions performed significantly worse than controls on multiple cognitive tasks—the TMT-A, TMT-B, MoCA, and UFOV divided attention. No differences emerged between IS with left-sided lesions (*n* = 6) across and driving or cognitive variables. The second analysis among SAH patients showed that those with MCA aneurysms (*n* = 5) spent a significantly greater distance out of the driving lane compared to controls and performed significantly worse, cognitively, on the MoCA and TMT-A. No differences emerged between controls and patients with anterior communicating artery aneurysms (*n* = 6). Taken together, these results suggest that IS patients with right-sided lesions and SAH patients with MCA aneurysms may be at particular risk for difficulty in driving performance, particularly lane maintenance, potentially related to subtle cognitive changes. Despite these promising findings, the sample size in this preliminary analysis was quite small and results should be interpreted with caution.

Across all stroke patients (IS + SAH), no cognitive tests were associated with driving outcomes. Variability in cognitive associations emerged across the stroke subtypes. Among patients with IS, the TMT-A, TMT-B, UFOV divided attention, and UFOV selective attention were significantly associated (*r*_s_ > 0.6, *p* < 0.05) with difficulty in lane maintenance (percentage distance out of the legal driving lane); however, these measures were not associated with total errors, turning errors, or speed maintenance (percentage distance over the speed limit) among patients with IS. No cognitive tests were associated with driving performance among patients with SAH. Similarly, a few previous studies have supported the use of the TMT (15, 19) and UFOV (15, 20, 21) in predicting driving performance among patients with stroke, whereas others report that these same measures offer no utility (22, 23). These results combined with the results of the current study suggest that the TMT and UFOV may offer some utility in predicting driving performance after stroke, particularly among those with IS; however, given the inconsistencies reported across studies, including the current study, no cognitive test has shown the sensitivity and specificity required to be implemented in a clinical setting.

There are a few limitations that warrant acknowledgment. High within-group variability was observed for both the IS and SAH groups, which is likely a factor contributing to the lack of significant finding in the IS (centerline crossings range = 0–32; left turn + traffic errors range = 0–8) as well as the SAH (collisions range = 0–12) groups. This reinforces that variability post-stroke extends beyond cognitive and neurological outcomes to one of the most complex functional tasks—driving. In addition, the current study included a relatively small sample size and did not include patients with intracerebral hemorrhage. Although in our preliminary analysis, we looked at a couple important clinical characteristics, we did not account for many other clinical characteristics and categories (e.g., bilateral lateralization, IS location, other SAH territories beyond anterior communicating artery and MCA, time since onset, severity of cognitive impairment, and so on). Given the heterogeneous presentation of stroke, it would be important for future research to include other stroke subtypes (e.g., intracerebral hemorrhage) as well as a larger sample size to account for other stroke characteristics (e.g., a variety of locations, size of lesion, severity of cognitive impairment, and functional ability) in addition to stroke subtype. In particular, previous driving studies across multiple neurological populations have emphasized the importance of executive function for safe driving (24, 25). Given this, coupled with the prevalence of executive dysfunction post-stroke, a more comprehensive examination of the influence of difficulties in executive function on driving ability post-stroke would be an important area of future research.

## Conclusion

Both individuals with IS and SAH exhibited difficulty with lane maintenance. Patients with IS additionally exhibited difficulty with speed maintenance, whereas SAH patients exhibited difficulty turning performance, particularly during left turns with traffic. Although most patients demonstrated safe driving behavior or minor difficulties, 30% exhibited impaired simulated driving behavior. This is concerning, as patients after stroke often do not receive driving advice from healthcare professionals or receive any form of driving evaluation (26, 27). Preliminary results suggest that IS patients with right-sided lesions and SAH patients with MCA aneurysms may be at particular risk for driving difficulty, particularly with lane maintenance; however, future large-scale work is required to confirm and expand on these findings. The TMT and UFOV may offer some utility in predicting driving performance among patients with IS; however, more research is required to determine the clinical utility of these measures. Thus, there are no tools with adequate sensitivity or specificity to assist healthcare professionals in determining driving safety. The current results highlight the importance of physicians discussing driving with their patients after stroke, both acutely and at follow-up, and investigating the driving ability, as well as their cognitive correlates, of different subtypes of stroke separately.

## Ethics Statement

This study was carried out in accordance with the recommendations of the Research Ethics Board at St. Michael’s Hospital with written informed consent from all participants. All participants gave written informed consent in accordance with the Declaration of Helsinki and the protocol was approved by the Research Ethics Board at St. Michael’s Hospital.

## Author Contributions

MH contributed to the study design, participant testing, data extraction, data analysis, data interpretation, drafting, and revising the work. KV contributed to the study design, participant testing, data extraction, data analysis, drafting, and revising of the work. TT contributed to data extraction and analysis, data interpretation, and revising the work. GS and RM contributed to the study design, participant recruitment, data interpretation, and drafting and revising of the work. TS contributed to the study design, data analysis, data interpretation, and drafting and revising of the work.

## Conflict of Interest Statement

LM is an employee and the chief scientific officer of Edge Therapeutics, Inc.
